# Fermented *Aloe arborescens* Miller Leaf Extract Suppresses Acute Alcoholic Liver Injury via Antioxidant and Anti-Inflammatory Effects in C57BL/6J Mice

**DOI:** 10.4014/jmb.2211.11044

**Published:** 2023-01-27

**Authors:** Min Ju Kim, Joon Hurh, Ha-Rim Kim, Sang-Wang Lee, Hong-Sig Sin, Sang-Jun Kim, Eun-mi Noh, Boung-Jun Oh, Seon-Young Kim

**Affiliations:** 1 Jeonju AgroBio-Materials Institute, Jeonju 54810, Republic of Korea; 2 Chebigen Co., Ltd., Jeonju 54853, Republic of Korea

**Keywords:** *Aloe arborescens* miller, fermentation, acute ethanol-induced liver injury, antioxidant, anti-inflammation

## Abstract

This study confirmed the change in functional composition and alcohol-induced acute liver injury in *Aloe arborescens* after fermentation. An acute liver injury was induced by administration of ethanol (3 g/kg/day) to C57BL/6J mice for 5 days. A fermented *A. arborescens* Miller leaf (FAAL) extract was orally administered 30 minutes before ethanol treatment. After fermentation, the emodin content was approximately 13 times higher than that of the raw material. FAAL extract significantly attenuated ethanol-induced aspartate aminotransferase, alanine aminotransferase, and triglyceride increases in serum and liver tissue. Histological analysis revealed that FAAL extract inhibits inflammatory cell infiltration and fat accumulation in liver tissues. The cytochrome P450 2E1, superoxide dismutase, and glutathione (GSH), which involved in alcohol-induced oxidative stress, were effectively regulated by FAAL extract in serum and liver tissues, except for GSH. FAAL also maintained the antioxidant defense system by upregulating heme oxygenase 1 and nuclear factor erythroid 2-related factor 2 protein expression. In addition, FAAL extract inhibited the decrease in alcohol dehydrogenase and aldehyde dehydrogenase activity, which promoted alcohol metabolism and prevented the activation of inflammatory response. Our results suggest that FAAL could be used as a potential therapeutic agent for ethanol-induced acute liver injury.

## Introduction

Alcohol abuse and excessive drinking lead to alcoholic liver diseases (ALDs), such as fatty liver, hepatitis, and cirrhosis. The morbidity and mortality of ALD are on the rise and are a global problem to be solved [1, 2].

Alcohol is absorbed in the stomach and small intestine, but most of its metabolism occurs in the liver [3]. Ethanol is oxidized to acetaldehyde by alcohol dehydrogenase (ADH) and then to acetate by acetaldehyde dehydrogenase (ALDH) in the liver [2]. Alanine transaminase (ALT) helps convert proteins into energy for the liver cells and Aspartate transaminase (AST) is an enzyme that supports metabolize amino acids. When the liver is damaged by alcohol, both enzymes are released into the bloodstream and levels increase [4, 5]. Oxidative stress is a key factor in the pathogenesis of ALD. Cytochrome P450 2E1 (CYP2E1) oxidizes alcohols to produce reactive oxygen species (ROS), such as superoxide and hydroxyl radicals, that cause oxidative stress [6, 7]. There are various compensatory mechanisms, including superoxide dismutase (SOD) and glutathione (GSH), to prevent liver damage caused by ROS [7].

Alcohol consumption disrupts intestinal permeability and activates nuclear factor-κB (NF-κB) signaling to promote the overproduction of proinflammatory cytokines. Cyclooxygenase-2 (COX2) and inducible nitric oxide synthase (iNOS) are enzymes involved in inflammatory processes. Studies have demonstrated the expression of COX2 and iNOS through the activation of NF-κB [8, 9]. Toll-like receptor 4 (TLR4) is a well-known family of TLR proteins. Signaling through TLR4 and myeloid differentiation primary response 88 (MyD88) mediates activation of NF-κB and subsequent production of proinflammatory cytokines including IL-1β and IL-6 [9, 10].

Leaf of *A. arborescens* Miller has long been used as a medicinal herb to treat diseases with various effects, including laxative, immune activation, and the ability to treat skin disorders [11]. *Aloe* leaf is divided into the rind and parenchyma, and each part is rich in anthraquinone glycosides and carbohydrates [12]. Anthraquinone derivatives, such as aloin, are converted into *Aloe*-emodin by intestinal esterase enzymes [11, 13-15]. *Aloe*-emodin exhibits many pharmacological effects, including anti-inflammatory, antibacterial and hepatoprotective activity [14-16].

The fermentation process increases the content of these bioactive compounds and converts the glycosides into more readily absorbable aglycones [17]. Recent studies have shown that fermented *A. arborescens* Miller has various physiological activities, such as hepatoprotective, antioxidant, and anti-wrinkle effects [18, 19]. To the best of our knowledge, there have been no reports on the changes in the content of bioactive compounds in extracts of fermented *A. arborescens* Miller leaf (FAAL) and the effects of FAAL extract on alcoholic liver damage. In this study, we fermented *A. arborescens* Miller leaf (AAL) with *Lactobacillus plantarum* JBMI F5, analyzed the *Aloe*-emodin content, and investigated its effect and signaling mechanism on alcohol-induced acute liver injury in mice.

## Materials and Methods

### Drugs and Reagents

Ethanol was obtained from Merck (Germany). Silymarin was obtained from Sigma Aldrich (USA). AST, ALT, ADH, and ALDH activity assay kits were obtained from BioVision (USA). Triglyceride (TG), ethanol, and catalase assay kits were purchased from Abcam (UK). Mouse IL-1β and IL-6 ELISA kits were purchased from R&D Systems Inc. (USA). A cytochrome P450 2E1 (CYP2E1) ELISA kit was purchased from Cusabio Technology (USA). A SOD assay kit was obtained from Biomax (Korea). A GSH assay kit was purchased from Cayman Chemical (USA). The primary and secondary antibodies used for western blotting were purchased from Cell Signaling Technology Inc. (USA) and Santa Cruz Biotechnology Inc. (USA).

### Preparation of FAAL

*Lactiplantibacillus plantarum* (*L. plantarum*) JBMI F5 (KACC91638P) was grown on MRS (de Man, Rogosa, and Sharpe) broth (Difo, USA) at 37°C for 16 h. AAL powder, which is removed the inedible parts (root and stem) of *A. arborescens* Miller, was purchased from Kim Jeong Moon *Aloe* Co., Ltd (Korea) and 100 g of AAL powder was extracted with distilled water (1:10, w/v) at 100°C for 24 h. The water extract of AAL was adjusted to pH 6.0 and sterilized at 121°C for 15 min. The water extract of AAL (pH 6.0) was inoculated with 5% (v/v) of *L. plantarum* (1 × 10^8^ cells/ml) and fermented for 16 h at 37°C in a shaking incubator (LSI-3016R, LabTech, Korea). At the end of fermentation, the pH of culture medium should be less than pH 4.0. The pH during fermentation was determined using a pH meter (BP3001, Trans instruments, Singapore). The FAAL suspension was lyophilized using a freeze dryer (MG-VFD300, MG industrial Inc., Korea) and stored at 4°C.

### Aloe-Emodin Quantification in AAL and FAAL Using High-Performance Liquid Chromatography (HPLC)

*Aloe*-emodin was quantified using an Agilent 1200 series HPLC equipped with a photodiode-array detector (Agilent Co., Ltd., USA). *Aloe*-emodin was solubilized in DMSO (1 mg/ml) and subsequently diluted in 70%aqueous methanol to prepare a series of standards ranging from 1-100 μg/ml. AAL and FAAL was solubilized in 70% aqueous methanol (50 mg/ml). The chromatographic separation was performed on a Gemini NX C18 column (4.6 × 250 mm, 5 μm i.d.; Phenomenex Inc., USA) at 25°C. The mobile phase consisted of deionized water containing a 0.5% acetonitrile (Solvent A) and acetonitrile (Solvent B) and was eluted using the following gradient: A/B (v/v) ratios of 90:10 for 0-10 min, 70:30 for 11-40 min, and 90:10 for 41-50 min. At a flow rate of 1.0 ml/min, a volume of 15 μl was injected into the HPLC instrument. *Aloe*-emodin was detected at 254 nm.

### Animals

Male C57BL/6J mice (20 - 25 g) were obtained from Damul Science (Korea). Mice were maintained under standard experimental conditions (22 ± 2°C, 55 ± 5%) with a 12 h light/dark cycle. The experimental protocol was carried out in accordance with the guidelines of the Animal Care Committee of Jeonju AgroBio-Materials Institute (IACUC JAMI2021004).

### Ethanol-Induced Acute Liver Injury in C57BL/6J Mice

The ethanol-induced acute liver injury mouse model was established with slight modifications of previous methods [20]. After acclimation for one week, the mice were randomly divided into 5 groups (5 mice in each group): N (normal, sterilized water), E (ethanol), PC (positive control, 200 mg/kg of silymarin), EAL (150 mg/kg body weight of AAL) and EFAL (150 mg/kg body weight of AAL fermented with 1.2 × 10^9^ CFU of LP). All experimental groups except N group were orally administered with ethanol once a day for 5 days 30 min before the assigned treatment. Body weight was measured at the same time every day. Blood and liver were collected after the last ethanol administration followed by a 12-h fast. Serum was obtained by centrifugation at 845 ×*g* for 15 min and then 13,523 ×*g* for 10 min. The serum and dissected liver were stored at -80°C until further analysis.

### Analysis of Liver Index and Serum Biomarkers

The liver index was calculated as the percentage of liver weight relative to body weight. ALT, AST, and TG were analyzed according to the manufacturer’s instructions.

### Histological Analysis

The liver tissues were fixed in 4% paraformaldehyde and embedded in paraffin. The tissue was cut into 4 μm sections and stained with hematoxylin and eosin (H&E). For liver lipid analysis, frozen liver sections were stained with Oil Red O solution. The stained tissue slides were observed and photographed using an optical microscope (Olympus, Japan).

### Detection of Alcohol Metabolism-Related Enzymes

The levels of alcohol metabolism-related enzymes were determined according to the instructions provided in the kits. The ethanol content was measured. Briefly, serum (50 μl) and reaction mixture (50 μl) were mixed in a 96-well plate, incubated for 30 min, and measured at 570 nm. ADH and ALDH were measured according to the instructions provided in the reagent kit.

### Determination of CYP2E1, SOD, and GSH Levels

CYP2E1, SOD, and GSH levels were measured according to the manufacturer’s protocols.

### Evaluation of IL-1β and IL-6

The levels of IL-1β and IL-6 in serum were evaluated by ELISA kits according to the manufacturer’s instructions.

### Western Blotting Analysis

Liver tissues were homogenized in lysis buffer containing a protease inhibitor cocktail. Protein concentrations were determined using a bicinchoninic acid protein assay kit. Proteins (20 μg) were resolved on a 10% SDS-polyacrylamide gel and then transferred to polyvinylidene difluoride (PVDF, GE Healthcare, UK) membranes. The blots were incubated with specific primary antibodies of HO-1, Nrf2, iNOS, COX2, TLR4, MyD88, p-IκBα, and p-NF-κB (1:2,500 dilutions) overnight at 4°C. After washed with TBST, the blots were incubated with respective species-specific horseradish peroxidase-conjugated secondary antibodies (1:5,000 dilutions) for 1 h at 25 ± 2°C. The bands were analyzed using densitometric scanning (Amersham imager 600, GE Healthcare) and quantitated using ImageJ 1.52 software (National Institutes of Health, USA).

### Statistical Analysis

Data are presented as the mean ± standard deviation (SD) derived from at least three separate experiments. One-way analysis of variance with Tukey’s post hoc test was performed to compare the parameters among the three groups, and Student’s *t*-test was used to assess the differences between the groups. *p* < 0.05 was considered statistically significant.

## Results

### Analysis of Aloe-emodin Contents in AAL and FAAL

**Fig. 1:**
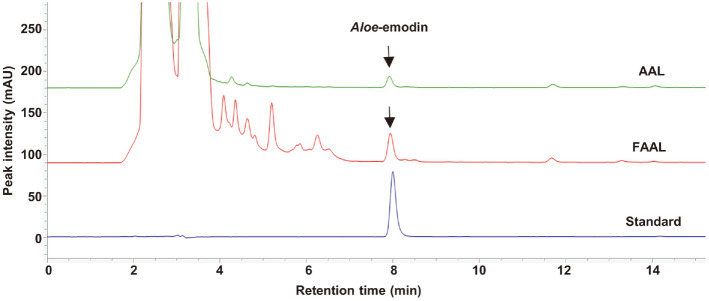
Analysis of *Aloe*-emodin in AAL and FAAL using high performance liquid chromatography (HPLC). HPLC chromatograms of AAL (upper), FAAL (middle), and *Aloe*-emodin standard (lower).

We used HPLC to analyze the change of *Aloe*-emodin content after AAL fermentation. As shown in Table 1 and Fig. 1, *Aloe*-emodin was markedly increased in FAAL (17.86 ± 0.24 mg/g) compared to AAL (1.32 ± 0.01 mg/g).

### Effects of AAL and FAAL on Body Weight Loss and Liver Injury

**Fig. 2:**
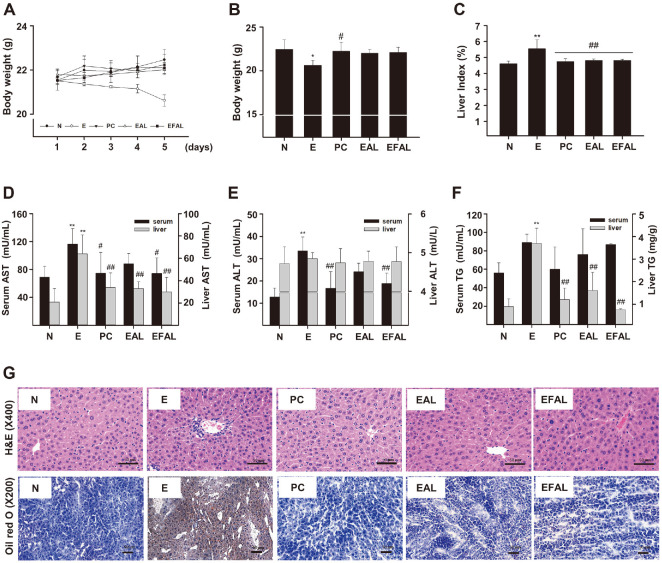
Effect of EAL and EFAL on ethanol-induced liver injury mice. (**A**) Changes in body weight of mice from 1 to 5 day. (**B**) Body weight of mice at 5 days. (**C**) The ratio of liver to body weight (liver index). (**D-F**) AST, ALT and TG levels in mouse serum and liver tissues. (**G**) Hematoxylin and eosin- and Oil Red O-stained sections of liver tissue. The observed sections were imaged at 400x and 200x magnification using a microscope. N (normal, sterilized water), E (ethanol), PC (positive control, 200 mg/kg of silymarin), EAL (150 mg/kg body weight of AAL) and EFAL (150 mg/kg body weight of FAAL). Data are given as the mean ± SD (*n* = 5). *p < 0.05, **p < 0.01 vs. the N group; ^#^*p* < 0.05, ^##^*p* < 0.01 vs. the E group.

AAL and FAAL were administered to mice for 5 days to investigate its effect on acute alcohol liver injury, respectively. As shown in Fig. 2A, the body weight of the mice in E group gradually decreased as the days went by, and significantly prevented (20.62 ± 0.26 g, *p* < 0.05) at the end of the experiment compared to the N group (22.46± 0.48 g) (Fig. 2B). Compared to the E group, the body weight loss by ethanol administration was recovered in the EAL and EFAL groups, respectively and there were no significant differences among these groups (Fig. 2B). The ratio of liver to body weight (liver index) was also markedly increased in the E group, but was significantly blocked in the EAL and EFAL groups, respectively (4.81 ± 0.09%, *p* < 0.01; 4.82 ± 0.07%, *p* < 0.01) (Fig. 2C).

The levels of serum AST and ALT were decreased in the PC, EAL, and EFAL groups compared to the E group (Figs. 2D and 2E). And the decrease of the serum levels by FAAL treatment was more effective than that by AAL treatment. Meanwhile, the levels of serum TG were not significantly different among the E, PC, EAL, and EFAL groups (Fig. 2F). In liver tissues, AST, and TG levels (64 ± 17 mU/ml, *p* < 0.01;3.69 ± 0.68 mg/g, *p* < 0.01) were significantly recovered by AAL and FAAL treatments, respectively (Figs. 2D and 2F). But ALT levels were not different among the E, PC, EAL, and EFAL groups (Fig. 2E).

Representative liver histological features are shown in Fig. 2G. The groups with ethanol administration showed slight pathological changes compared to the N group. The groups were characterized by increase lipid vacuoles and inflammatory cell infiltration. These kinds of ethanol-induced histopathological changes were prevented by AAL and FAAL treatments, respectively (Fig. 2G, upper panel). Oil Red O is soluble in lipids bound to triglycerides, so it can be used to analyzed fat accumulation in liver tissue. As shown by staining liver tissues with Oil Red O, lipid droplets were found in the ethanol-induced groups, but were decreased in the EAL and EFAL groups (Fig. 2G, lower panel). These results suggest that AAL and FAAL effectively prevent alcohol-induced liver injury and lipid accumulation.

### Effects of AAL and FAAL on Alcohol Metabolism

**Fig. 3:**
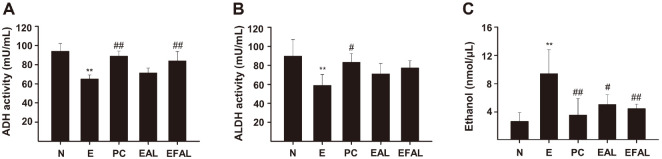
Effect of EAL and EFAL on alcohol metabolism-related enzymes in ethanol-induced liver injury mice. (**A-C**) ADH and ALDH activitys, and ethanol contents in the serum of the acute ethanol-induced mouse model. Data are given as the mean ± SD (*n* = 5). *p < 0.05, **p < 0.01 vs. the N group; ^#^*p* < 0.05, ^##^*p* < 0.01 vs. the E group.

As shown in Figs. 3A and 3B, ADH and ALDH activities were significantly decreased in the E group (65.09 ± 4.235 mU/ml, *p* < 0.001; 59.02 ± 11.235 mU/ml, *p* < 0.01) compared to the N group. And the ethanol-induced enzyme inactivation was alleviated by AAL and FAAL treatments, respectively. The ADH level in the EFAL group was similar to that in the PC group (84.06 ± 9.659 mU/ml, *p* < 0.01) (Fig. 3A). In addition, ethanol administration increased the blood ethanol concentration, which was significantly inhibited by AAL and FAAL treatments, respectively (5.08 ± 1.37 nmol/μl, *p* < 0.05; 4.5 ± 0.609 nmol/μl, *p* < 0.01) (Fig. 3C). These results suggest that AAL and FAAL regulate alcohol metabolism, and that the fermentation with LP may enhance the effects of AAL on alcohol metabolism.

### Antioxidant Effect of AAL and FAAL in Alcohol-Induced Liver Injury

**Fig. 4:**
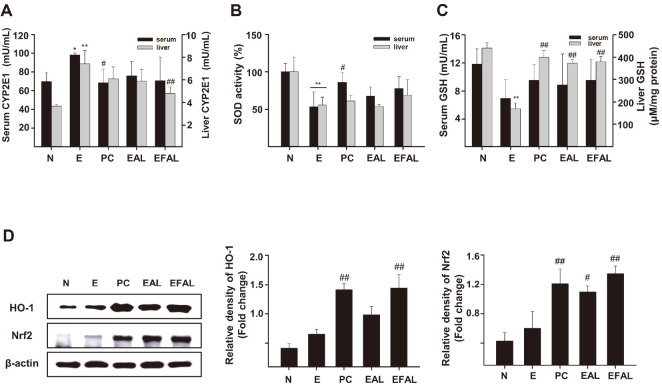
Effect of EAL and EFAL on antioxidant activity in ethanol-induced liver injury mice. (**A-C**) Activity levels of CYP2E1, SOD activity, and GSH content in the serum and liver tissue of an acute ethanol-induced liver injury mouse model. (**D**) Expression levels of antioxidant-related proteins (HO-1 and Nrf2) in liver tissues of the acute ethanol-induced mouse model. Data are given as the mean ± SD (*n* = 5). *p < 0.05, **p < 0.01 vs. the N group; ^#^*p* < 0.05, ^##^*p* < 0.01 vs. the E group.

Acute and chronic alcohol treatment increases ROS production and stimulates oxidative stress in various biological systems. CYP2E1 is known to play an important role on alcohol-induced oxidative stress in the liver by generating ROS during alcohol metabolism [6]. The levels of CYP2E1 were significantly increased in the serum and liver tissues of E group (98.48 ± 1.37 mU/ml, *p* < 0.05; 7.41 ± 1.20 mU/ml, *p* < 0.01) (Fig. 4A). The enhancement was inhibited in both serum and liver tissues by AAL and FAAL treatment, respectively. The EFAL group was found to be the most effective in alleviating the CYP2E1 increase in liver tissues caused by ethanol (4.78± 0.6 mU/ml, *p* < 0.01) (Fig. 4A).

SOD, GSH and HO-1 are antioxidant genes promoted by the transcription factor Nrf2, and are representative defense system that protect hepatocytes from ROS [6]. The SOD activity was significantly reduced in both serum and liver tissues in the E group (53.16 ± 19.76%, *p* < 0.01; 55.67 ± 10.2%, *p* < 0.01) compared to the N group (Fig. 4B). On the other hand, alcohol-induced decrease in SOD activity was alleviated in both EAL and EFAL groups. And the effect was stronger in the EFAL group than in the EAL group. The GSH levels was also significantly decreased in both serum and liver tissues of the E group compared to the N group (Fig. 4C). The reduction was restrained by AAL and FAAL treatment, respectively.

To further evaluate the antioxidant effect of AAL and FAAL, we performed a western blot for the expression of antioxidant-related proteins. The EAL and EFAL groups showed significantly an increased expression of HO-1 and Nrf2 compared to the E group (Fig. 4D). These results suggest that AAL and FAAL can prevent alcohol-induced liver damage by inhibiting oxidative stress through HO-1/Nrf2 protein up-regulation.

### AAL and FAAL Attenuate Inflammation through Modulation of TLR4/NF-κB Signaling

**Fig. 5:**
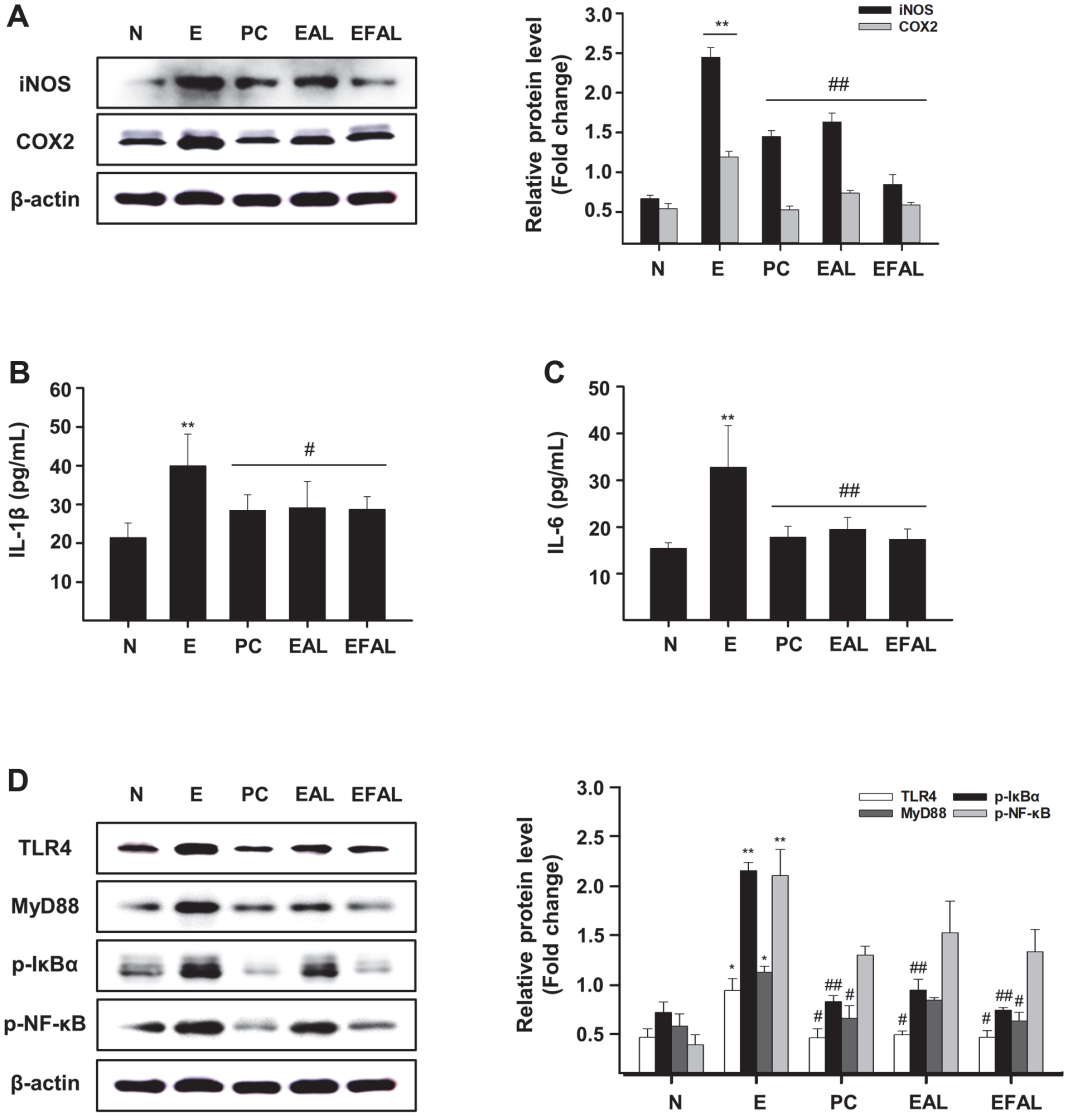
EAL and EFAL suppressed inflammation in an acute ethanol-induced liver injury mouse model. (**A**) Expression levels of inflammation-related proteins (COX2 and iNOS) in liver tissue of the acute ethanol-induced liver injury mouse model. (**B** and **C**) The levels of IL-1β and IL-6 in the serum of the acute ethanol-induced liver injury mouse model. (**D**) Expression of the TLR4/MyD88/NFκB signaling pathway in liver tissues of the acute ethanol-induced mouse model. Data are given as the mean ± SD (*n* = 5). *p < 0.05, **p < 0.01 vs. the N group; ^#^*p* < 0.05, ^##^*p* < 0.01 vs. the E group.

To elucidate the anti-inflammatory effects of AAL and FAAL in mice with acute alcohol intake, we measured the expression of iNOS and COX2 proteins in the livers of mice. The expression levels of iNOS and COX2 proteins were significantly higher in the E group than in the N group (Fig. 5A). In contrast, iNOS and COX2 protein levels in the liver were significantly lower in the EAL and EFAL groups than in the E group. The inflammatory cytokines IL-1β and IL-6 were evaluated for their anti-inflammatory effects on alcohol-induced liver inflammation. Serum IL-1β and IL-6 levels were significantly higher in the E group (40.01 ± 8.07 pg/ml, *p* < 0.01; 32.81 ± 8.91 pg/ml, *p* < 0.01) than in the N group, and their levels were decreased by AAL (29.19 ± 6.81 pg/ml, *p* < 0.05; 19.5 ± 2.52 pg/ml, *p* < 0.01) and FAAL (28.8 ± 3.22 pg/ml, *p* < 0.05; 17.35 ± 2.22, *p* < 0.01) treatment, respectively (Figs. 5B and 5C).

TLR4/MyD88 and NF-κB which are signaling molecules implicated in alcohol-induced liver injury were further investigated on the protective effects of AAL and FAAL. The expression of TLR4 and the resulting molecule MyD88 in the liver was significantly higher in the E group than in the N group (Fig. 5D). And the expression of TLR4 and MyD88 in the livers was significantly decreased in the EAL and EFAL groups compared to in the E group.

Moreover, we investigated another downstream signaling target of TLR4/MyD88, p-IκBα and p-NF-κB. In particular, the expression of p-IκBα and p-NF-κB in the liver was significantly higher in the E group than in the N group (Fig. 5D). Significant reductions in both p-IκBα and p-NF-κB were observed in the livers of mice in the EAL and EFAL groups compared to the E group. These results indicate that AAL and FAAL attenuate alcohol-induced hepatic inflammation by regulating the TLR4/MyD88/NF-κB signaling pathway.

## Discussion

ALD is a leading cause of morbidity and mortality worldwide [1, 2]. The most prominent factor in the pathogenesis of ALD is free radical-mediated oxidative stress [7].

*Aloe* plant has been used as a skin disease treatment for a long time [11]. Recently, it has been reported to have various therapeutic effects, such as antioxidant and anti-inflammation effects [21]. Among the active compounds in *Aloe*, *Aloe*-emodin has been reported to have various pharmacological effects, including hepatoprotective, anti-inflammatory, and anticancer effects [14, 15].

In our results, fermentation of AAL resulted in an approximately 13-fold increase in *Aloe*-emodin content. Moreover, we found that AAL fermentation enhanced the protective effect against liver injury in a mouse model.

When liver cells are damaged by acute alcohol ingestion, the structural integrity of the liver is impaired, resulting in increased cell membrane permeability. As a consequence, AST and ALT in the cytoplasm are released into the blood circulation, so they are used as indicators to evaluate liver function [4, 5]. TG is mainly metabolized in the liver and is widely used as an indicator of fatty liver because it accumulates in adipocytes in the early stage of ALD [22]. Previous study [23] demonstrated that A. *vera* polysaccharides exert a potent inhibitory effect on chronic alcohol-induced levels of ALT, AST and TG in a mouse model. In this study, we showed that AAL and FAAL significantly alleviated the increase in AST, ALT, and TG than the only ethanol administration group, and that FAAL had a stronger effect than AAL (Figs. 2D and 2F). These results suggest that FAAL is effective in mitigating acute ethanol-induced liver injury and fatty liver in mice.

Alcohol is oxidized to acetaldehyde by ADH and then to acetic acid by ALDH in the liver [2]. However, excessive alcohol intake activates the microsomal ethanol oxidizing system and induces CYP2E1 expression and alcohol metabolism by CYP2E1 to generate ROS and inhibits the activity of antioxidant enzymes, leading to oxidative stress. Many CYP2E1 inhibitors have been reported to inhibit ALD, so it is considered to be a target for the treatment for ALD [6]. In our study, alcohol intake significantly decreased ADH and ALDH levels and increased CYP2E1 and blood ethanol levels (Figs. 3 and 4A). Treatment with AAL and FAAL improved these factors related to alcohol metabolism, and FAAL was more effective. These results suggest that AAL and FAAL have the ability to improve alcohol metabolism.

Oxidative stress is a major mechanism of alcohol-induced liver damage [6, 7]. Nrf2, a transcription factor associated with the antioxidant system [24], promotes various antioxidant genes, such as GSH, SOD, and HO-1, in oxidative stress reactions. These antioxidant genes are representative antioxidant defense systems that protect hepatocytes from ROS [25, 26]. Since *Aloe* plant contains a large amount of polysaccharides and flavonoids, it has antioxidant activity by inhibiting the formation of free radicals and ameliorates acute colitis via the Nrf2/HO-1 signaling pathway [27]. Our results showed that AAL suppressed the decrease of SOD activity and GSH levels in serum and liver tissues caused by acute ethanol administration (Figs. 4B and 4C). And AAL increased the expression of HO-1 and Nrf2 in liver tissue (Fig. 4D). In addition, these effects were further increased by FAAL. These are consistent with previous study that *Aloe* fermentation increases antioxidant activity [18], indicating the efficacy of AAL and FAAL on antioxidant signaling. Taken together, we suggest that the antioxidant capacity of FAALs improves alcohol metabolism, and is involved in hepatoprotective effects.

In addition, *Aloe* plant is known to be effective in various inflammatory conditions, such as allergies and wound healing. Among them, it has been reported that *Aloe*-emodin has an anti-inflammatory effect by modulating iNOS and COX2 in arthritis [28]. We found that AAL and FAAL treatment significantly prevented alcohol-induced increases in hepatic iNOS and COX2 protein expressions, and FAAL more effectively protected alcohol-induced iNOS and COX2 expression (Fig. 5A).

Excessive alcohol consumption induces the release of proinflammatory cytokines by activating the TLR4/MyD88/NF-κB signaling pathway that causes liver inflammation [10]. It was also reported that alcohol-induced liver damage was suppressed in TLR4-deficient mice [29], and aloe emodin inhibits TLR4 expression in the inflammatory response through hydrophilic interaction with ARG-322, an active residue of TLR4 [30].

Consistently, our results also showed that alcohol induced the activation of TLR4/MyD88 and phosphorylation of IκBα and NF-κB, leading to increased inflammatory factors such as iNOS, COX2, IL-1β, and IL6. FAAL suppressed the activation of these signaling pathways and the increase of inflammatory factors by alcohol. In addition, FAAL prevented oxidative stress by promoting ethanol metabolism, activating HO-1 and Nrf2, and inhibiting CYP2E1 expression.

Our findings demonstrate that FAAL effectively alleviates acute alcohol-induced liver injury by inhibiting oxidative stress and inflammatory responses. This suggests that FAAL could be a potential treatment for acute ethanol-induced liver injury.

## Figures and Tables

**Table 1:** *Aloe*-emodin content in AAL and FAAL.

Sample	Contents (mg/g)	RSD (%)
AAL	1.32 ± 0.01	0.8
FAAL	17.86 ± 0.24	1.3

AAL, *A. arborescens* Miller leaf; FAAL, Fermented AAL with 5% (v/v) *Lactobacillus plantarum* JBMI F5; RSD, relative standard deviation. Values are the means ± SD of three independent experiments.
